# Alpha-Tomatine Induces Apoptosis and Inhibits Nuclear Factor-Kappa B Activation on Human Prostatic Adenocarcinoma PC-3 Cells

**DOI:** 10.1371/journal.pone.0018915

**Published:** 2011-04-26

**Authors:** Sui-Ting Lee, Pooi-Fong Wong, Shiau-Chuen Cheah, Mohd Rais Mustafa

**Affiliations:** Department of Pharmacology, Faculty of Medicine, University of Malaya, Kuala Lumpur, Malaysia; Universita di Sassari, Italy

## Abstract

**Background:**

Alpha-tomatine (α-tomatine) is the major saponin in tomato (*Lycopersicon esculentum*). This study investigates the chemopreventive potential of α-tomatine on androgen-independent human prostatic adenocarcinoma PC-3 cells.

**Methodology/Principal Findings:**

Treatment of highly aggressive human prostate cancer PC-3 cells with α-tomatine resulted in a concentration-dependent inhibition of cell growth with a half-maximal efficient concentration (EC_50_) value of 1.67±0.3 µM. It is also less cytotoxic to normal human liver WRL-68 cells and normal human prostate RWPE-1 cells. Assessment of real-time growth kinetics by cell impedance-based Real-Time Cell Analyzer (RTCA) showed that α-tomatine exhibited its cytotoxic effects against PC-3 cells as early as an hour after treatment. The inhibitory effect of α-tomatine on PC-3 cancer cell growth was mainly due to induction of apoptosis as evidenced by positive Annexin V staining and decreased in mitochondrial membrane potential but increased in nuclear condensation, polarization of F-actin, cell membrane permeability and cytochrome c expressions. Results also showed that α-tomatine induced activation of caspase-3, -8 and -9, suggesting that both intrinsic and extrinsic apoptosis pathways are involved. Furthermore, nuclear factor-kappa B (NF-κB) nuclear translocation was inhibited, which in turn resulted in significant decreased in NF-κB/p50 and NF-κB/p65 in the nuclear fraction of the treated cells compared to the control untreated cells. These results provide further insights into the molecular mechanism of the anti-proliferative actions of α-tomatine.

**Conclusion/Significance:**

α-tomatine induces apoptosis and inhibits NF-κB activation on prostate cancer cells. These results suggest that α-tomatine may be beneficial for protection against prostate cancer development and progression.

## Introduction

Prostate cancer is one of the leading causes of deaths in men worldwide with major mortality due to advanced stage of the cancer where androgen-independent cells become unresponsive to hormone ablation therapy [1], [2]. There is a pressing need to identify alternative chemopreventive measures as surgery and current chemotherapeutic strategies can be ineffective against the aggressive form of prostate cancer [3]–[5].

Failure of cells to undergo apoptotic cell death contributes to the development of cancers, hence treatment that induces apoptosis would be a promising anticancer strategy [6], [7]. Natural products are important sources of new and less toxic anticancer agents. A variety of dietary agents such as resveratrol, curcumin, sulforaphane, gingerol, indole-3 carbinol, withanolide and green tea catechins can induce apoptosis and are effective against various human cancer cells tested [7]–[11]. These also have an added advantage of lesser toxicity compared to current standard therapeutic drugs [12].

The present work investigates the chemopreventive potential of alpha-tomatine (α-tomatine) against prostate cancer cells. α-tomatine is the major saponin in tomato (*Lycopersicon esculentum*). It possesses antimicrobial, antifungal, anti-inflammatory and immunopotentiating activities [13]–[18]. It is protective against dibenzo[a,l]pyrene (DBP)-induced liver and stomach tumors in rainbow trout [19] and also promotes anti-proliferative effects against human colon HT-29, liver HepG2, breast MCF-7 and stomach AGS cancer cells [20], [21]. Thus far, it is only known that α-tomatine acts on phosphoinositide 3-kinase and protein kinase B (PI3K/Akt) and extracellular signaling-regulating kinase (ERK) signaling pathways in lung adenocarcinoma A549 cells [22]. The present study, hence, seeks to investigate the effect of α-tomatine on the growth of human prostatic adenocarcinoma PC-3 cells and to identify its potential growth inhibitory mechanism. Results from the present study would provide new insights into the beneficial role of α-tomatine for prevention of prostate cancer development and progression.

## Materials and Methods

### Phytochemicals, standard drug and reagents

α-tomatine (purity>97%) was purchased from Extrasythese (Genay, France). Paclitaxel from Ascent Scientific (Weston-SuperMare, UK), a standard drug used in chemotherapy regimen for prostate cancer was used as positive control in this study. Curcumin (Merck, Germany) was used as positive control in NF-κB translocation assay. Dimethyl sulfoxide (DMSO) and tumor necrosis factor-alpha (TNF-α) were purchased from Sigma Aldrich (St. Louis, MO). Both the tested compound and positive controls were prepared in 100% DMSO, stored at−20°C, and then diluted as needed in cell culture medium. Penicillin/streptomycin, Dulbecco Modified Eagle Medium (DMEM), Roswell Park Memorial Institute (RPMI 1640), keratinocyte growth medium, fetal bovine serum (FBS) and MTT (3-(4,5-dimethylthiazol-2-yl)-2,5-diphenytetrazolium bromide) were purchased from Invitrogen (Carlsbad, CA).

### Cell lines

Prostate cancer PC-3 (CRL-1435), normal human liver WRL-68 (CL-48) cells, and normal human prostate RWPE-1 (CRL-11609) were obtained from the American Type Culture Collection (Manassas, VA) and cultured in RPMI-1640, DMEM and keratinocyte growth medium, respectively. Both RPMI-1640 and DMEM were supplemented with 10% heat-inactivated FBS, 100 U/mL penicillin and 100 µg/mL streptomycin. Cells were cultured at 37°C in a humidified atmosphere of carbon dioxide–air (5∶95).

### *In vitro* cytotoxicity screening

Briefly, adherent cells were seeded in sterile 96-wells plates at optimum cell density. After 18–24 hours, cells were treated with increasing concentrations (0.16–5.0 µM) of α-tomatine for 24 hours. Cells treated with 0.2% DMSO was used as vehicle control. Following treatment, cells were incubated in the dark with 2 mg/ml MTT at 37°C for 2 hours, then the medium was carefully removed and 100 µl of DMSO was added to dissolve the formazan crystals formed. Absorbance was measured at 570 nm in a plate reader. Cell viability was calculated using the following formula: (mean absorbance in test wells)/(mean absorbance in control well)×100%. Dose-response curves were plotted using GraphPad Prism 4 (GraphPad Software Inc., San Diego, CA). Half maximal effective concentration (EC_50_) values were determined using non-linear regression model with sigmoidal dose response (variable slope) following the algorithm of GraphPad Prism 4.

### Real time cell proliferation analysis

Real time growth kinetics of PC-3 cells was examined by impedance-based Real-Time Cell Analysis (RTCA) system (Roche Diagnostic, Mannheim, Germany). RTCA utilizes E-plate which contains inter-digitated micro-electrodes on the bottom of the plate that detect local ionic changes as cells proliferate and is measured as electrode impedance. Briefly, 50 µl of medium was added in 16-wells E-plate and background readings were recorded. Cell suspension (50 µl) at cell density of 1.25×10^4^ cells/well was added to each well of the E-plate. The attachment, spreading and proliferation of the cells were monitored every 5 minutes intervals. When the cells entered logarithmic growth phase, they were treated with 100 µl of α-tomatine at various concentrations and continuously monitored every 10 minutes for up to 72 hours. Cells treated with 0.2% of DMSO was used as vehicle control and monitored in parallel with the α-tomatine and paclitaxel-treated cells. Cell sensor impedance was expressed as an arbitrary unit called the Cell Index (CI). The CI at each time point was defined as (R_n_−R_b_)/15, where R_n_ is the cell-electrode impedance of the well and the R_b_ is the background impedance of the well with the media alone. Growth curves were normalized to the CI at the last measured time point before compound addition for each well.

### Annexin V/propidium iodide (PI) double staining assay

Apoptosis-mediated cell death of tumor cell was examined by a double staining method using FITC-labeled Annexin V/PI apoptosis detection kit (BD Bioscience, San Jose, CA) according to the manufacturer's instructions. Briefly, control and α-tomatine-treated cells were collected, washed in cold phosphate-buffered saline (PBS) twice, stained with fluoresceinisothiocyanate (FITC)-conjugated Annexin V and PI dyes. The externalization of phoshotidylserine and the permeability to PI were evaluated by FACS Calibur flowcytometer (BD Bioscience, San Jose, CA). Data from 10,000 gated events per sample were collected. Cells in early stages of apoptosis were positively stained with Annexin V; whereas, cells in late apoptosis were positively stained with both Annexin V and PI.

### Multiparametric High Content Screening (HCS) assays

HCS (Cellomics Inc, Pittsburgh, PA, USA) enables concurrent quantitative measurement of multiple independent cellular phenotypes using fluorescent probes. Both Cellomics multiparametric cytotoxicity and apoptosis HitKit™ (Pittsburgh, PA, USA) were used to examine cellular changes in the α-tomatine-treated PC-3 cells. Multiparametric cytotoxicity HitKit™ contains four fluorescent dyes, i.e. blue fluorescent Hoechst 33342, membrane permeability, mitochondrial membrane potential, and cytochrome c dyes. They respectively detect changes in nuclear morphology (nuclear condensation), membrane permeability, mitochondrial membrane potential and expression of cytochrome c. The multiparametric apoptosis kit quantifies three fundamental parameters related to the process of apoptosis, i.e. (i) nuclear condensation, detected by the blue fluorescent nuclear dye, Hoechst 33342; (ii) F-actin content, detected by green fluorescent Alexa Fluor ® 488Phalloidin stain; (iii) mitochondrial membrane potential, based on the uptake of MitoTracker ® Red into mitochondria of cells. Briefly, PC-3 cells were seeded overnight at density of 8000 cells/well into flat-bottomed 96-well plates (Perkin-Elmer Inc., Wellesley, MA, USA). Following treatment with different concentrations of α-tomatine (0.25 to 2.0 µM), fixation and staining for imaging analysis of the PC-3 cells were performed according to the manufacturer's instructions. Cells treated with 0.2% DMSO and 5.0 µM paclitaxel were used as negative and positive controls, respectively. Plates were analyzed using Thermo Scientific ArrayScan®VTI HCS Reader (Cellomics Inc, Pittsburgh, PA, USA). This is a computerized automated fluorescence imaging microscope that automatically identifies stained cells and measures the intensity and distribution of fluorescence in individual cells. Images for each fluoroprobe were acquired at different channels using suitable filters with 20× objective at fixed exposure time. The Cell Health Profiling BioApplication software was used for image acquisitions and analysis. For each well, at least 25 fields, corresponding to at least 500 cells were automatically acquired and analyzed. All experiments were performed in triplicates. Cell average intensity (Mean) under the modified object mask within selected range in each channel was used as assay indicator, and reported as average fluorescence intensity.

Cell cycle phase distribution of α-tomatine-treated cells was determined using Cellomics Cell Cycle BioApplication as described previously [23]. It applies similar principles as FACS in assessing cell cycle distribution where the intensity of Hoechst stained nucleus is deemed proportional to the cell's DNA content. Total fluorescence intensity of the dye from the nucleus of each cell typically exhibits a bimodal distribution. The first peak typically contains cells with 2N DNA content (G_0_/G_1_ phase), the second peak with a double intensity of the first, contains cells with 4N DNA content (G_2_/M phase). Under normal conditions, there are more cells in the G_0_/G_1_ versus the G_2_/M phase. The region between these two peaks represents cells with DNA content between 2N and 4N (S phase) which are cells in the process of doubling their DNA. Cells found with DNA<2N distribution are usually apoptotic cells. To identify the range of each of the DNA content categories, the software first identifies 2N (G_0_/G_1_ phase) and 4N (G_2_/M phase) DNA content peaks using vehicle control cells. Then, it automatically classified each cell's nuclear total intensity into one of the categories of DNA content: sub G_1_ (DNA<2N), G_0_/G_1_ (DNA∼2N), S (2N<DNA>4N) and G_2_/M (DNA∼4N) phases in α-tomatine-treated cells. The percentage of each cell cycle phase for vehicle control, paclitaxel-treated cells and α-tomatine-treated cells were then reported.

### Caspase activity

The activities of caspase-3, -8 and -9 were measured using the fluorometric assay kit (Calbiochem, USA) following the protocol of the manufacturer. Briefly, cells were treated with α-tomatine (2.0 µM) with or without inhibitors (caspase-8: z-IETD-FMK; caspase-9: z-LEHD-FMK; caspase-3-like: DEVD-CHO). After treatment, the cells were harvested using trypsinization and cell lysates were prepared as described [24]. The cell lysates were mixed with reaction buffer and 10 µl of fluorogenic peptide substrate: Ac-DEVD-AMC (caspase-3), Ac-IETD-AMC (caspase-8) and Ac-LEHD-AMC (caspase-9), and incubated for 2 hours at 37°C in the dark. Inhibitors were added 30 minutes before addition of fluorogenic substrate. Wells containing 50 µl of sample buffer, 50 µl of assay buffer and 10 µl of substrate were used as blank. Purified caspase was used as positive control while untreated cell extract was used as negative control. Fluorescence was then measured at excitation of 390 nm and emission of 500 nm. Fold-increase in the protease activity was determined by comparing the levels of the treated cells with untreated controls.

### NF-κB translocation assay

NF-κB translocation in PC-3 cells was examined using NF-κB activation HCS kit which contains Hoechst 33342 and Alexa Fluor 488 conjugated anti-NF-κB dyes. PC-3 cells were seeded into sterile flat-bottomed 96-well plates (Perkin-Elmer Inc., Wellesley, MA, USA) at 8000 cells/well (100 µl/well). After 18–24 hours, cells were treated with different concentrations of α-tomatine for 30 minutes, followed by treatment with 10 ng/ml TNF-α for another 30 minutes. Cells pre-treated with 0.2% DMSO and 50 µM curcumin were used as negative and positive inhibitor controls, respectively. Fixation, permeabilization and immunofluorescence staining of cells were performed according to the manufacturer's instructions. For target translocation analysis, cells stained with Hoechst dye were identified as objects in channel 1 and an adjustable mask called Circ was created around every nucleus. In non-activated cells, Alexa Fluor 488-stained NF-κB was detected in channel 2 in the cytoplasm where an annular region called Ring was defined beyond the nuclear region. Images were acquired using suitable filters with 20× objective at fixed exposure times. For each well, at least 500 cells were automatically acquired and analyzed. The output feature MEAN_CircRingAvgIntenDiffCh2 represents the difference between the intensity of nuclear and cytoplasmic NF-κB associated fluorescence (Nuc-Cyto Diff), reported as translocation parameter, as previously described [25].

### NF-κB/p50 and NF-κB/p65 transcription factor assay

PC-3 cells at 70–80% confluence were treated with α-tomatine for 30 minutes, followed by treatment with TNF-α (10 ng/ml) for another 30 minutes. The cells were then washed with PBS and the both nuclear and cytoplasmic fractions of the treated cells were extracted using nuclear extraction kit (Cayman Chemical, Ann Arbour, MI). Concentrations of the active forms of NF-κB/p50 and NF-κB/p65 in the both fractions were measured using Cayman NF-κB/p50 and NF-κB/p65 ELISA kits (Ann Arbour, MI) according to the instructions of the manufacturer. The differences in NF-κB/p50 and NF-κB/p65 levels between the nuclear and cytoplasmic fractions were reported.

### Statistical analysis

All assays were conducted in at least three separate experiments. Results are expressed as the mean value ± standard deviation (SD). Statistical analysis was performed with one-way analysis of variance (ANOVA), with Dunnett's Multiple Comparison Test to identify between-group differences using GraphPad Prism software (version 4.0; GraphPad Software Inc., San Diego, CA). Statistical significant is expressed as ***, p<0.001; **, p<0.01; *, p<0.05. Log EC_50_ calculations were performed using the built-in algorithms from dose-response curves with variable slope.

## Results

### α-tomatine dose-dependently inhibited proliferation of PC-3 cancer cells

**Figure 1:**
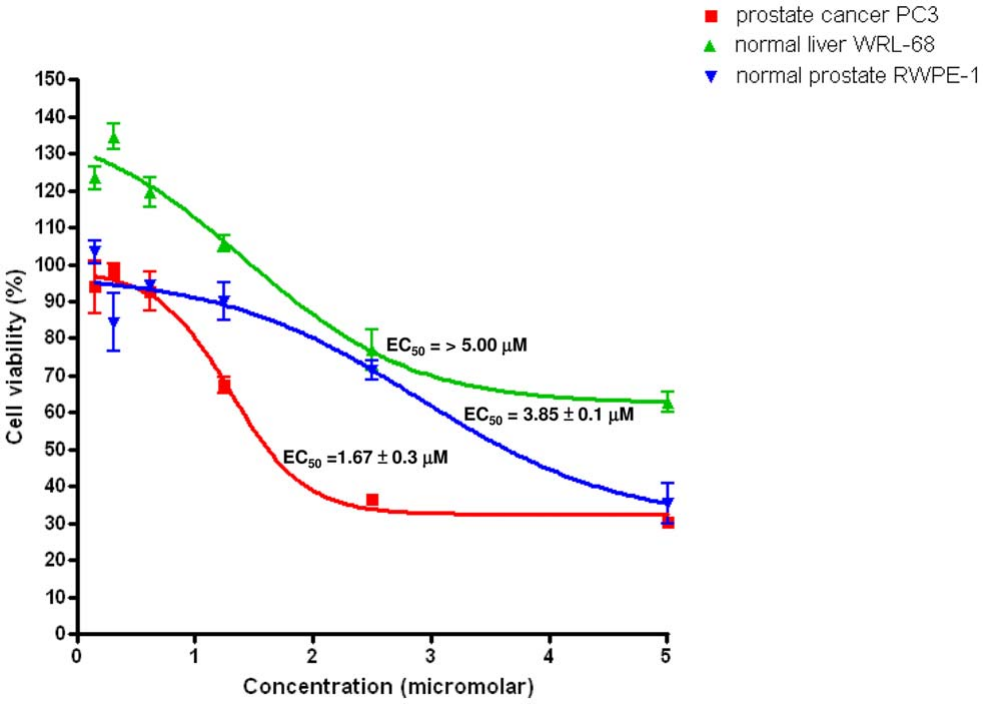
The effect of α-tomatine on cell viability of PC-3, WRL-68 and RWPE-1 cells. Cells were treated with α-tomatine for 24 hours and cell proliferation was measured using MTT reduction assay. Each value represents the mean ± SD of three independent experiments, performed in triplicates. EC_50_ values of α-tomatine on PC-3 cancer cell, WRL-68 normal cells and RWPE-1 normal prostate cells at 24 hours treatment were 1.67±0.3 µM, >5.0 µM and 3.85±0.1 µM, respectively.

To evaluate the effect of α-tomatine on cell viability of PC-3, WRL-68 and RWPE-1 cells, MTT assay was performed. Treatment of α-tomatine to PC-3 cells resulted in a significant dose-dependent (from 0.16 to 5.0 µM) inhibition of cell growth (Figure 1). The EC_50_ value at 24 hours post-treatment with α-tomatine for PC-3 cells was estimated at 1.67±0.3 µM. α-tomatine only caused cytotoxicity towards normal prostate RWPE-1 cells at highest concentration (5.0 µM) of α-tomatine, with EC_50_ value of 3.85±0.1 µM. Normal human liver WRL-68 cells treated with the highest concentration of α-tomatine tested (5.0 µM) resulted in ∼35% of nonviable cells and this suggest that α-tomatine is less cytotoxic against WRL-68 cells when compared to PC3 cells. The EC_50_ values of α-tomatine towards WRL-68 cells (>5 µM) and RWPE-1 (3.85±0.1 µM) and were higher than that of PC-3 cells (1.67±0.3 µM), suggesting the selective cytotoxicity of α-tomatine towards prostate cancer PC-3 cells.

### Real-time growth kinetics analysis of α-tomatine using cell impedance-based analyzer

**Figure 2:**
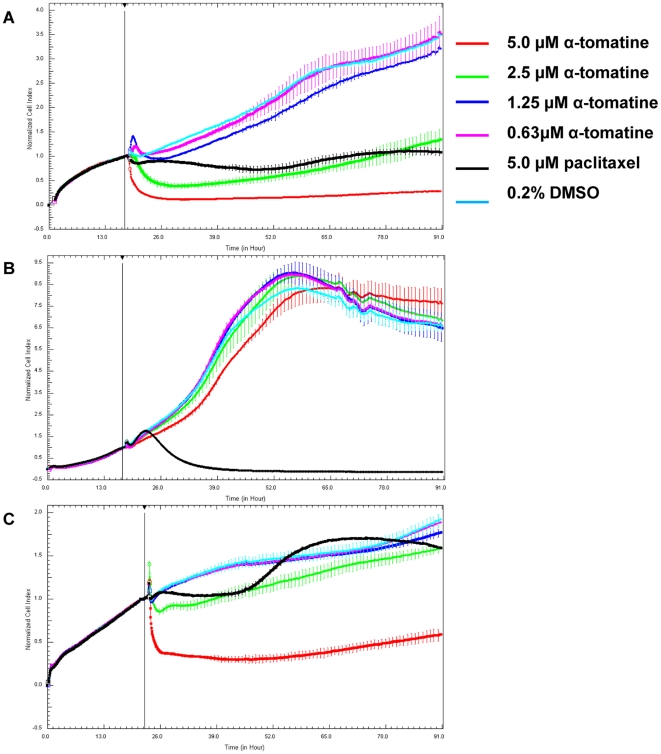
Dynamic assessment of cell viability after treatment with α-tomatine. Normalized Cell Index (CI) measured over 72 hours on A) prostate cancer PC-3, B) human normal liver WRL-68 and C) human normal prostate RWPE-1 cells. Briefly, cells at logarithmic growth phase were treated with α-tomatine and CI values were recorded every 10 minutes intervals using RTCA MP system. Cells treated with 5.0 µM paclitaxel and 0.2% DMSO were used as positive and negative controls, respectively. A change in impedance as the cells spread on the E-plate was displayed as CI value. Plotted CI values were normalized to the last time point before addition of treatment.

The dynamics of PC-3 cells proliferation on the 16-wells E-plates were monitored at every 5 minutes interval from the time of plating until the cells entered the logarithmic growth phase, following which the cells were treated with different concentrations of α-tomatine. After treatment, CI values were acquired at every 10 minutes interval for 72 hours. It was observed that a rapid decrease in CI value occurred as early as an hour after treatment with 5.0 µM and 2.5 µM of α-tomatine on PC-3 cells (Figure 2A), suggesting that PC-3 cells were dying from the treatment with these concentrations. PC-3 cells treated at lower concentrations (0.16–1.25 µM) of α-tomatine were proliferating in parallel to cells treated with vehicle control as indicated with increase in CI values. In contrast, WRL-68 cells treatment with α-tomatine showed no reduction in CI values even at the highest tested concentration of 5.0 µM compared to control levels and it exhibited a continuous rise in CI values throughout the 72 hours of treatment (Figure 2B). This showed that WRL-68 cells growth was not affected by α-tomatine. Treatment with 2.5 µM α-tomatine initially inhibited normal prostate RWPE-1 cell proliferation but eventually recovered to the level of vehicle control cells (Figure 2C). Treatment with higher concentration (5.0 µM), however, decreased the CI values suggesting that α-tomatine inhibited the proliferation of RWPE-1 cells at high concentration (Figure 2C). These data suggested that α-tomatine at low concentration (2.5 µM) can inhibit the growth of PC-3 prostate cancer cells but not the normal prostate RWPE-1.

### α-tomatine induces apoptosis on PC-3 cancer cells

**Figure 3:**
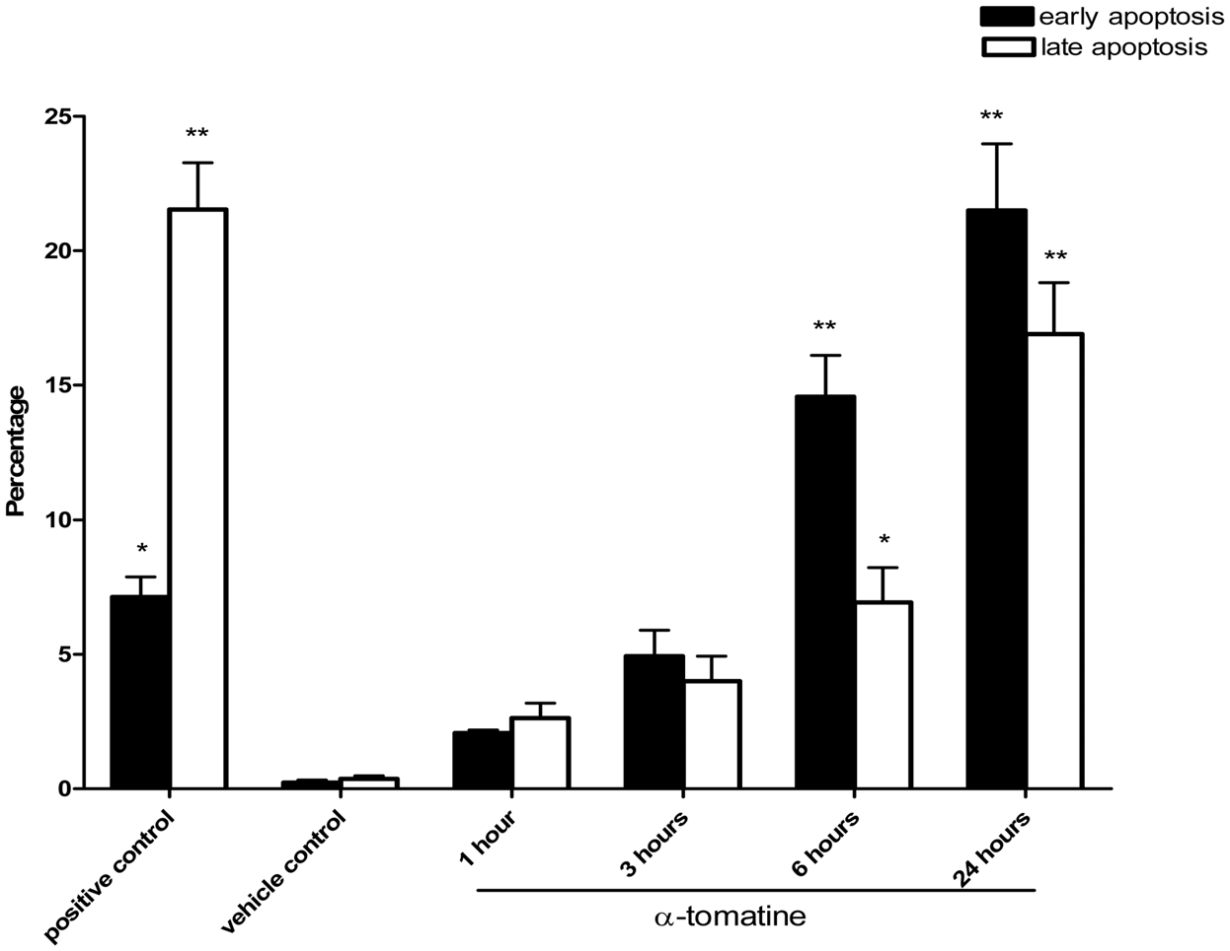
Annexin V/PI double staining assay. Cells were exposed to either 0.2% DMSO (vehicle control), 5.0 µM paclitaxel (positive control), or α-tomatine for the indicated time. The treated cells were stained with Annexin V and PI and then subjected to FACS analysis. The percentages of early apoptotic cells (Annexin V positive, PI negative) and late apoptotic cells (Annexin V and PI positive) observed at different incubation time with α-tomatine are shown. Results are presented as the mean ± SD of three independent experiments. * *P*<0.05, ** *P*<0.01, *** *P*<0.001 *vs* vehicle control.

To determine if α-tomatine induces apoptosis in PC-3 prostate cancer cells, Annexin V/propidium iodide (PI) double staining assay and flow cytometry analysis were performed. Treatment with 2.0 µM α-tomatine for 1, 3, 6 and 24 hours resulted in a gradual increase in early apoptotic cells (Annexin V positive only) from 2.07±0.12% to 5.00±0.97%, 14.57±1.55%, and 21.50±2.48%, respectively (Figure 3, black bars). The late apoptotic cells (Annexin V and PI positive) were also increased significantly, from 2.63±0.56% to 4.00±0.93%, 6.93±1.3%, and 16.9±1.92% (Figure 3, white bars). Positive control cells treated with 5.0 µM paclitaxel for 24 hours also resulted in 7.13±0.75% of early apoptotic cells (Figure 3, black bars) and 21.53±1.74% of late apoptotic cells (Figure 3, white bars) in the culture compared with only 0.23±0.09% of early apoptotic cells (Figure 3, black bars) and 0.37±0.12% of late apoptotic cells (Figure 3, white bars) in the negative control cells treated with 0.2% DMSO. Hence, these results showed that α-tomatine inhibited PC-3 cells growth by inducing apoptosis in these cells.

### Multiparametric HCS assays

**Figure 4:**
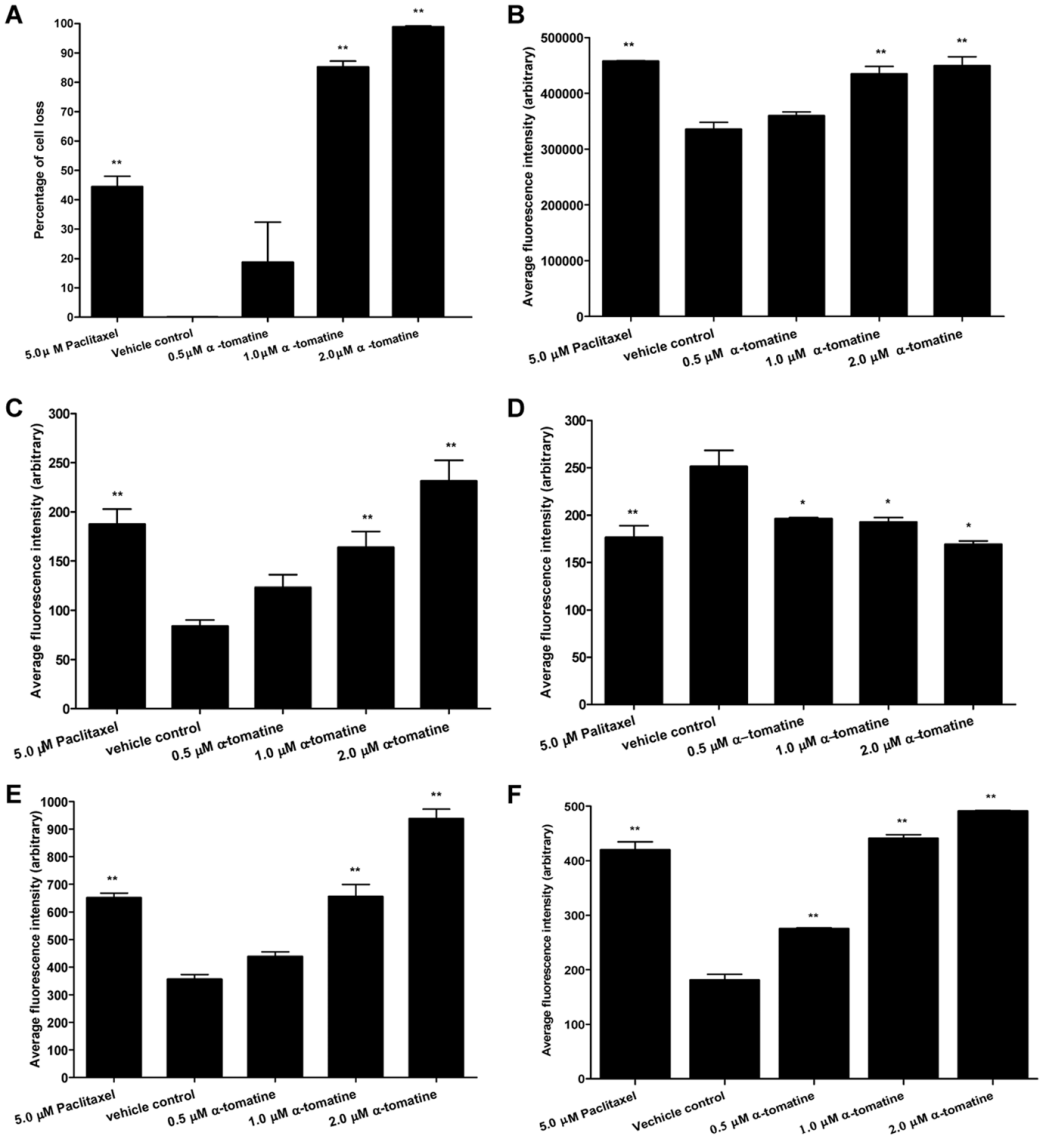
HCS analysis of apoptosis associated cellular morphology on α-tomatine-treated PC-3 cells. PC-3 cells were treated with different concentrations of α-tomatine (0.5–2.0 µM). Cell morphology changes associated with apoptosis such as A) percentage of dead cells, B) alterations in nuclear condensation, C) membrane permeability, D) mitochondrial membrane potential, E) expression of cytochrome c and F) F-actin contents are reflected by the average fluorescence intensity detected. Cells treated with 0.2% DMSO and 5.0 µM paclitaxel were used as negative and positive controls, respectively. All measured parameters were expressed as average fluorescent intensities and averaged for at least 500 cells per well. Data is representative of three independent experiments.* *P*<0.05, ** *P*<0.01, *** *P*<0.001 *vs* vehicle control.

**Figure 5:**
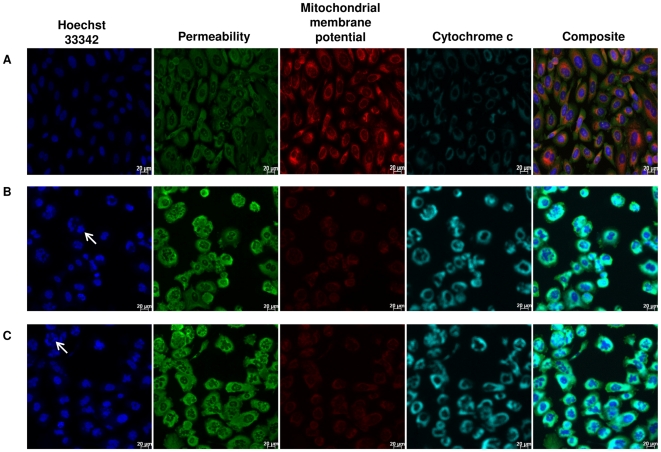
Cytotoxic and proapoptotic effects of α-tomatine on PC-3 cells. PC-3 cells were treated for 24 hours with A) 0.2% DMSO, B) 5.0 µM paclitaxel, and C) 2.0 µM α-tomatine. The treated cells were examined for nuclear condensation using blue Hoechst 33342 stain, changes in membrane permeability using a green fluoroprobe, mitochondrial membrane potential using MitoTracker Red CMXRos dye, and cytochrome c expression using a blue green fluoroprobe. Scale bar indicates 20 µm.

To further confirm the induction of apoptosis by α-tomatine, the treated cells were examined for cellular changes associated with apoptosis using HCS analysis. Treatment with 2.0, 1.0 and 0.5 µM α-tomatine resulted in approximately 95%, 83% and 20% cell loss, respectively (Figure 4A). In comparison, positive control cells treated with 5.0 µM paclitaxel resulted in only 45% cell loss and none from the vehicle control cells (Figure 4A). The α-tomatine treated cells also exhibited a concentration-dependent increase in nuclear chromatin staining with Hoechst 33342 (Figures 4B and 5C), suggesting an increase in nuclear condensation. Increased in nuclear chromatin staining with Hoechst 33342 was also observed in the paclitaxel positive control cells (Figure 4B and 5B) whereas the fluorescent intensity was significantly lower and healthy nuclear morphology was observed in the vehicle control cells (Figures 4B and 5A). Membrane permeability of the treated cells also increased significantly as evidenced by high fluorescent intensity in the cytoplasm of α-tomatine (Figure 4C and 5C) and paclitaxel- treated cells (Figure 4C, and 5B) compared to the vehicle control (Figures 4C and 5A). A reduction in mitochondrial membrane potential is observed in both paclitaxel (Figure 4D and 5B) and α-tomatine (Figure 4D and 5C) treated cells compared to intact mitochondrial membrane potential of the vehicle control cells (Figure 4D and 5A). More specific apoptosis indicators such as release of cytochrome c and F-actin polarization or cleavage were found increased in a concentration-dependent manner in the cytoplasm of PC-3 treated cells, with the highest increase observed at 2.0 µM α-tomatine (Figure 4E, 4F and 5C). These results further confirmed that α-tomatine inhibited PC-3 cell growth by inducing apoptosis.

### α-tomatine-induced apoptosis in PC-3 cells is not associated with cell cycle arrest

**Figure 6:**
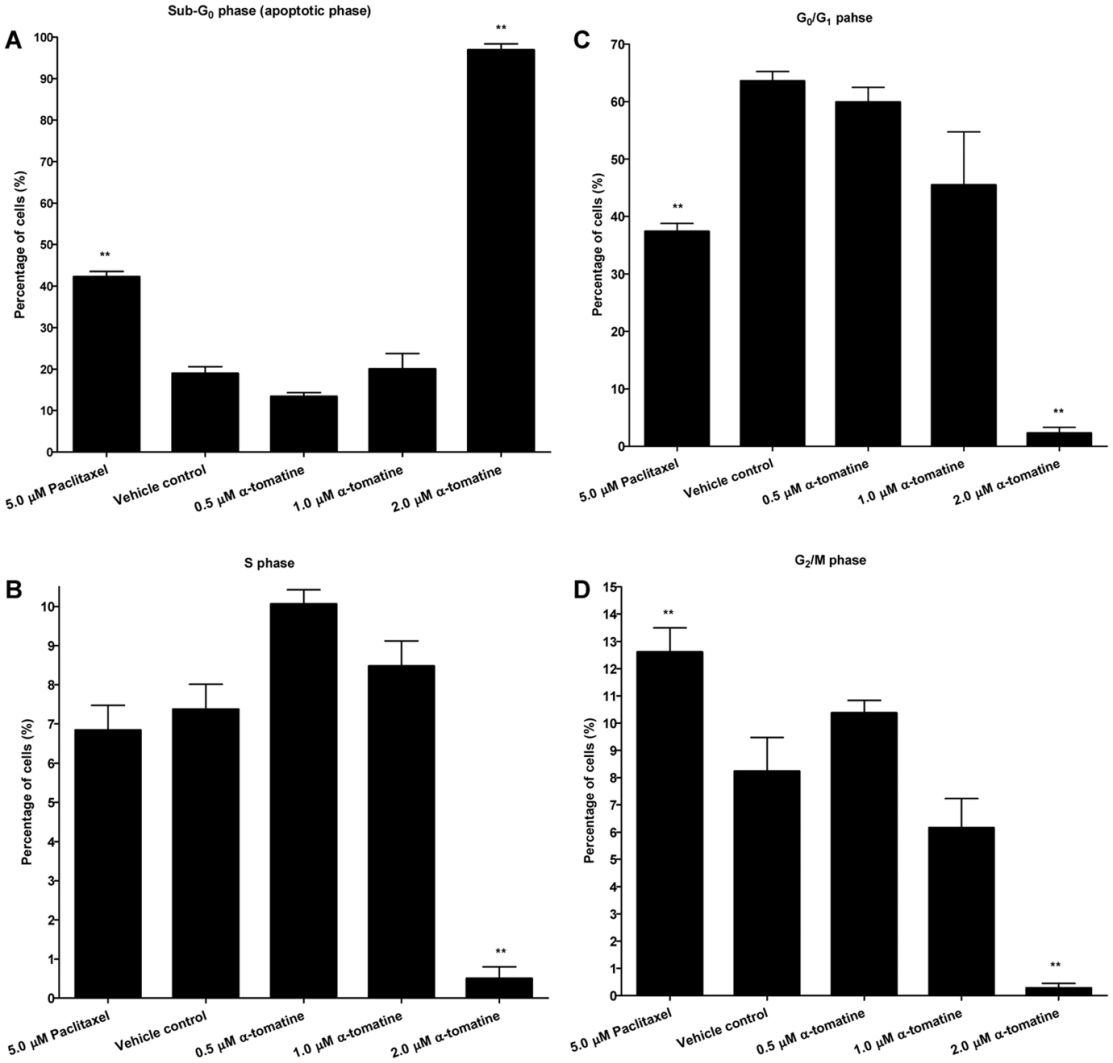
Cell cycle distribution of α-tomatine-treated PC-3 cells. The percentage of α-tomatine-treated cells at A) Sub G_1_, B) G_0_/G_1_, C) S, and D) G_2_/M phases of PC-3 cells. Each bar represents the mean ± SD of data from three independent experiments.* *P*<0.05, ** *P*<0.01, *** *P*<0.001 *vs* vehicle control.

To determine whether induction of apoptosis in PC-3 cells by α-tomatine could be related to the cell cycle arrest, cell cycle distribution of the treated cells was assessed by labelling the cell's DNA with blue fluorescent Hoechst 33342 stain, whose intensity is proportional to the cell's DNA content. The sub G_1_ population representing apoptotic cells in the 2.0 µM α-tomatine treated cells was markedly increased (95%) compared to positive control cells treated with paclitaxel (40%, Figure 6A). This increase was reflected by significant reduction in cells at G_0_/G_1_ (Figure 6B), S (Figure 6C), and G_2_/M phases (Figure 6D). No significant changes in the percentage of population of cells in other phases were observed in 0.5 and 1.0 µM treated cells, suggesting that induction of apoptosis was not mediated by cell cycle arrest.

### α-tomatine induced caspases activation

**Figure 7:**
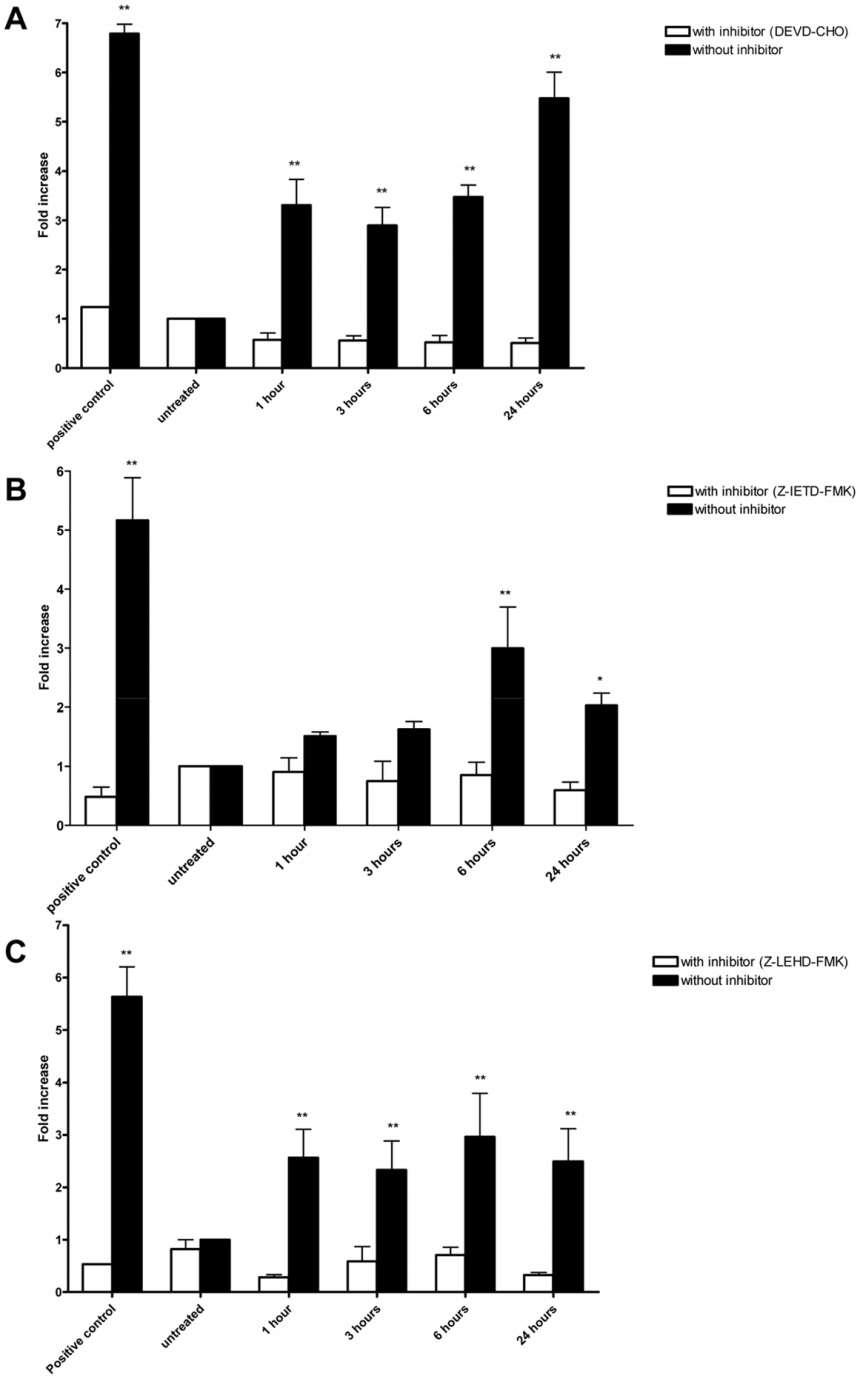
Effect of α-tomatine on caspases activation. Fold increase of the levels of A) caspase-3, B) caspase-8, and C) caspase-9 in PC-3 cells treated with 2.0 µM α-tomatine, compared to vehicle control. The caspase activities were determined in the presence or absence of specific inhibitors in time course manner. The fluorescence intensity was measured at excitation wavelength of 390 nm and emission wavelength of 500 nm. The increase of protease activities was determined by comparing the levels in α-tomatine-treated PC-3 cells with the vehicle control. Each bar represents the mean ± SD of data from three independent experiments.* *P*<0.05, ** *P*<0.01, *** *P*<0.001 *vs* vehicle control.

Caspases are present in the proforms (inactive) and become active after site-specific cleavage to participate in the process of apoptosis. To determine whether caspases are involved in apoptosis induction by α-tomatine, the protein levels of active caspases in α-tomatine-treated cells were evaluated. Activation of the executioner procaspase-3 by α-tomatine was found to be time-dependent (Figure 7A). Caspase-3 activity was significantly elevated at the first hour of treatment and progressed to a maximal level (6-fold over vesicle control) after 24 hours of incubation (Figure 7A). High levels of pro-caspase-8 and pro-caspase 9 were detected as early as 1 hour after the addition of α-tomatine, and reached a maximal level at 6 hours of incubation where the high levels of caspase 8 and 9 persisted over 24 hours of incubation (Figure 7B and 7C). These findings suggest that α-tomatine activated caspase-3, caspase-8 and caspase-9 as early as 1 hour after treatment. Cell permeable-specific inhibitors of caspase-3-like (DEVD-CHO, Figure 7A), caspase-8 (z-IETD-FMK, Figure 7B) and caspase-9 (z-LEHD-FMK, Figure 7C) were added into α-tomatine treatment to determine whether the activation of caspase-3, -8 and -9 by α-tomatine can be blocked by these inhibitors. In the presence of inhibitors, fold increase of caspase-3 (Figure 7A), caspase-8 (Figure 7B) and caspase-9 (Figure 7C) were reduced. These results further confirmed the activation of caspase-3, -8 and -9 by α-tomatine in PC-3 cells.

### α-tomatine inhibits TNF-α-induced NF-κB nuclear translocation

**Figure 8:**
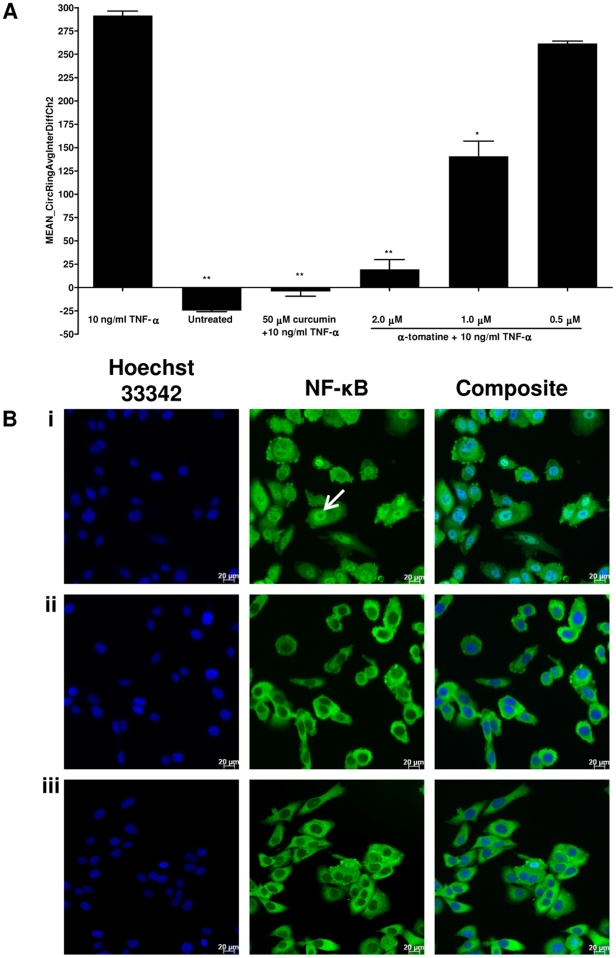
The inhibitory effect of α-tomatine on TNF-α-induced NF-κB nuclear translocation. PC-3 cells were pretreated with α-tomatine for 30 minutes and followed by 10 ng/ml of TNF-α stimulation for 30 minutes. A) Cells treated with 50 µM curcumin and medium only were used as positive NF-κB inhibitor and negative control, respectively. Cells were fixed, immunostained with anti-NF-κB antibody, and counterstained with Hoechst 33342. NF-κB nuclear translocation index was measured using Thermo Scientific ArrayScan®VTI HCS Reader and expressed as the difference between nuclear and cytoplasmic NF-κB related fluorescence intensity. Each bar represents the mean ± SD of data in triplicate.* *P*<0.05, ** *P*<0.01, *** *P*<0.001 *vs* cells treated withTNF-α alone. B) Images of PC-3 cells treated with 10 ng/mL TNF-α alone (i), medium only (ii), and 2.0 µM of α-tomatine pretreatment before 10 ng/ml TNF-α stimulation (iii). Control and treated PC-3 cells were stained with Hoechst (blue) and the Alexa Fluor 488-conjugated anti-NF-κB antibody (green). Images were acquired for each fluorescence channel, using suitable filters with 20× objective. Scale bar indicates 20 µm.

It has been shown that activation of NF-κB blocks apoptosis and promotes cell proliferation [26]. We assessed whether α-tomatine inhibits activation of NF-κB induced by the inflammatory cytokine, TNF-α using Alexa Fluor 488-conjugated anti-NF-κB antibody. In cells treated with medium only (Figure 8A and 8Bii), high fluorescent intensity of NF-κB was found in cytoplasm but dimly in nuclei, indicating that NF-κB was not activated under resting condition. Following stimulation with TNF-α alone, NF-κB fluorescent intensity significantly increased in nuclei, suggesting that TNF-α stimulation resulted in NF-κB activation and translocation from cytoplasm into nuclei occurred in the stimulated cells (Figure 8A and 8Bi). However, in PC-3 cells treated with curcumin, a known inhibitor of NF-κB activation, it was observed that significant inhibition of TNF-α-induced NF-κB nuclear translocation as evidenced by low nuclear NF-κB-related fluorescence intensity (Figure 8A). Similarly, α-tomatine also inhibited TNF-α-induced NF-κB activation in a dose-dependent manner with strong inhibition observed at 2.0 µM treatment (Figure 8A, and 8Biii). These results showed that α-tomatine inhibit NF-κB activation.

### α-tomatine treatment inhibited NF-κB/p50 and NF-κB/p65 nuclear translocation

**Figure 9:**
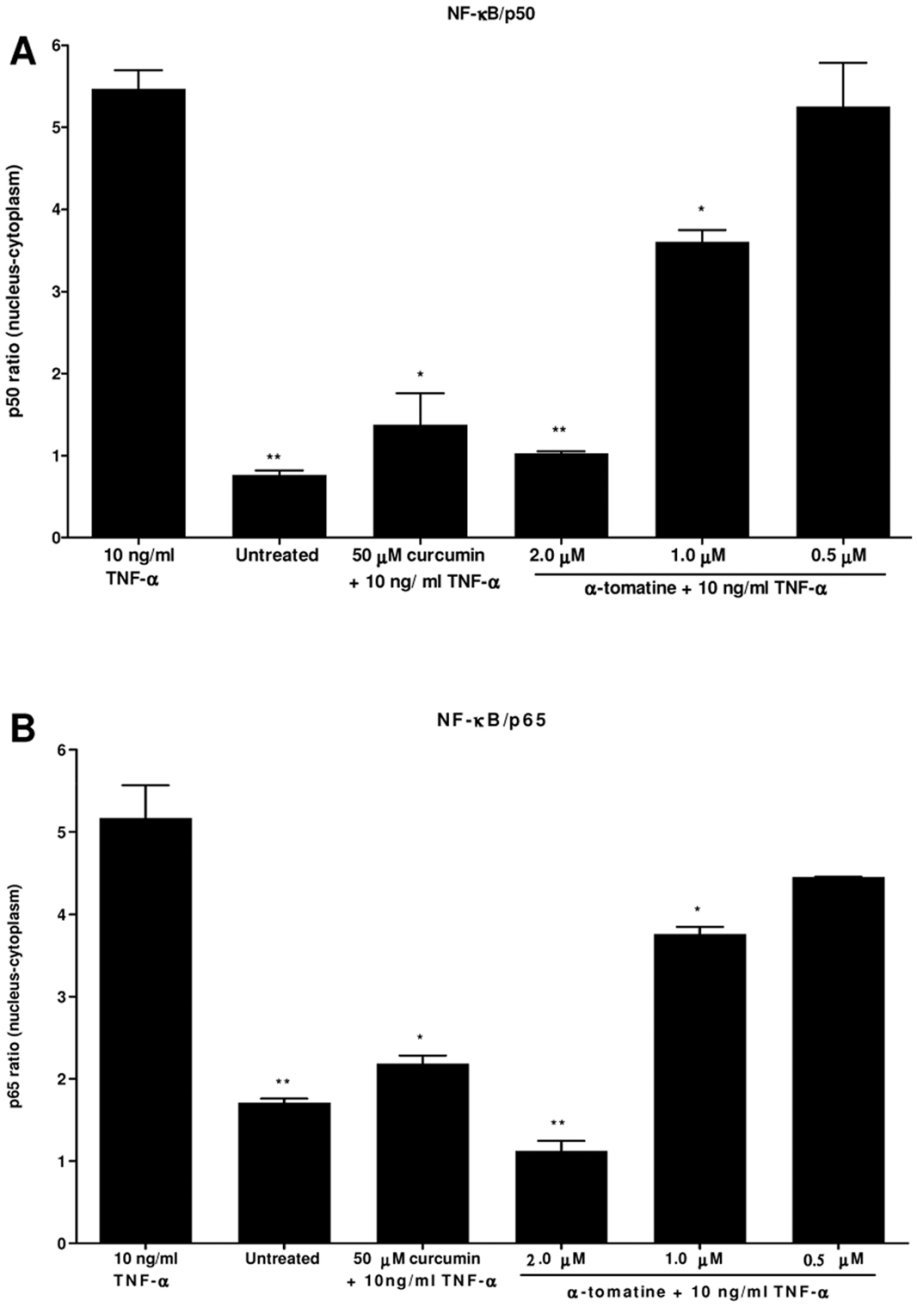
Comparison of NF-κB/p50 and NF-κB/p65 protein levels between nuclear and cytoplasmic fraction. PC-3 cells were treated with 0.5, 1.0, 2.0 µM α-tomatine for 30 minutes, followed by treatment with 10 ng/ml TNF-α for another 30 minutes. Nuclear and cytoplasmic fractions of the PC-3 treated cells were extracted, the concentration of the active form of A) NF-κB/p50 and B) NF-κB/p65 in both fractions were measured with ELISA kits. Differences of NF-κB/p50, NF-κB/p65 in the nuclear and cytoplasmic fractions are reported. Each bar represents the mean ± SD of data in triplicate.* *P*<0.05, ** *P*<0.01, *** *P*<0.001 *vs* cells treated with TNF-α control.

To further confirm TNF-α-induced NF-κB activation was inhibited in α-tomatine treated PC-3 cells through the inhibition of NF-κB nuclear translocation, NF-κB/p50 (Figure 9A) and NF-κB/p65 (Figure 9B) in nuclear and cytoplasmic fractions of the treated cells were measured by ELISA. Pretreatment with 2.0 µM of α-tomatine resulted in significant decreased in both NF-κB/p50 (Figure 9A) and NF-κB/p65 (Figure 9B) level in nuclear fractions, compared to cells treated with TNF-α control (Figure 9A and 9B). These results showed that α-tomatine inhibits TNF-α-induced NF-κB activation by inhibiting nuclear translocation of NF-κB/p50 and NF-κB/p65 in the treated PC-3 cells.

## Discussion

α-tomatine was reported to inhibit growth of human colon HT-29, liver HepG2, breast MCF-7 and stomach AGS cancer cells [20], [21] but its anti-proliferative mechanisms require further definition. The present study reports that α-tomatine inhibits NF-κB activation and induces apoptosis on androgen-independent human prostatic adenocarcinoma PC-3 cells. The EC_50_ of α-tomatine towards PC-3 cells was estimated at 1.67±0.3 µM and this value is comparable to earlier studies on different cancer cell lines [20], [21]. Dynamic assessment by xCelligence system showed that α-tomatine is a fast-acting compound, inhibiting the cancer cells proliferation as early as an hour after treatment with 2.5 and 5.0 µM α-tomatine and its growth suppressive effect was not fully reversible as the cancer cell proliferation failed to recover throughout 72 hours of treatment. It is also less cytotoxic against human liver normal cells and human prostate normal cells. Although our data showed that α-tomatine inhibited the growth of normal prostate RWPE-1 cells at high concentration (5.0 µM), where utilization of high dose of α-tomatine can be a key consideration for cancer treatment, earlier *in vivo* studies showed no apparent toxic effects in rainbow trout [19] and does not affect body and liver weights of mice [14], [27].

Flow cytometry analysis of Annexin V/PI staining showed that α-tomatine conspicuously induced apoptosis. This observation was confirmed by multiparametric cell-based high content screening (HCS) analysis that showed morphological features characteristic of apoptotic cell death such as nuclear condensation, loss of membrane symmetry, polarization of F-actin, release of cytochrome c and reduction in mitochondrial membrane potential. Disruption of the mitochondrial membrane potential is an early event in apoptosis and triggers release of cytochrome c and other apoptogenic molecules from the mitochondria to the cytosol [28]. These apoptogenic molecules contribute to the activation of caspases and subsequent cell death. More intensive molecular studies indicate that apoptotic cell death can be triggered either through receptor (extrinsic)-mediated pathway where the ligand-receptor binding activates caspase-8 or the mitochondrial (intrinsic)-mediated pathway where cytochrome c is released from the mitochondrial and activates caspase-9 [29], [30]. Both of these mechanisms will eventually merge and lead to the hierarchical activation of the downstream caspase-3, 6 and 7, which are responsible for the characteristic apoptosis-associated morphological changes such as chromatin condensation, membrane blebbing, and loss of overall cell shape [31], [32]. The present study demonstrated that treatment with α-tomatine increased activities of caspase-3 and -9 and caused depolarization of mitochondrial membrane potential and release of cytochrome c. These results suggest that induction of apoptosis is mediated through the intrinsic pathway. Pro-caspase-8 is an intracellular component that directly communicates with the death domain of cell membrane receptors and its activation in the α-tomatine-treated cells suggests that apoptosis is also mediated through the extrinsic pathway which perhaps merged with the intrinsic pathway and activated the executioner caspase 3. Consistent with these data, addition of cell permeable-specific inhibitors of caspase-8 (z-IETD-FMK), caspase-9 (z-LEHD-FMK), or caspase-3-like (DEVD-CHO) enzymes revealed that addition of caspase inhibitors completely decreased α-tomatine-induced activation of caspase-3, -8 and -9, confirmed the role of caspase-3, -8 and -9 in α-tomatine-induced apoptosis. Curcumin is an example of dietary agent that mediates apoptosis via both intrinsic and extrinsic pathways [33].

α-tomatine could also significantly alter polymerization of actin filaments in the treated PC-3 cells. This observation is in line with reports that showed polymerization or cleavage of actin cytoskeleton during the early and late phases of apoptosis [34]–[37]. Cisplatin, a well-known anticancer drug is an example of drug that induces F-actin damage prior to changes in nuclear morphology [38].

While the present study found no evidence of cell cycle arrest by α-tomatine, there are evidence that suggest induction of apoptosis could be mediated through the inhibition of nuclear factor-kappa B (NF-κB) signaling pathway. Constitutive NF-κB activation has been observed in many types of cancer, including androgen-independent prostate cancer [26]. Overexpression of NF-κB/p65 protein was found in the nuclear fraction of prostate cancer clinical specimens [39], [40], suggesting the pathophysiological role for NF-κB in prostate cancer progression. Constitutive activation of NF-κB in tumor cells could promote cancer cells survival by blocking apoptosis and promote cancer cells growth through angiogenesis, metastasis and invasion [41]. Accumulating evidence indicate that constitutive activation of NF-κB in tumor cells always contribute to chemoresistance and radioresistance, and represents an independent risk factor for recurrence after radical prostatectomy [40], [42], [43]. Hence, targeting the NF-κB signaling pathway remains an attractive therapeutic option for prostate cancer. Treatment of PC-3 cells with α-tomatine resulted in a strong inhibition of NF-κB activation, which was consistent with a decrease in nuclear levels of NF-κB/p65 and NF-κB/p50. The ability of α-tomatine in inhibiting NF-κB activation by blocking the nuclear translocation of NF-κB/p65 and NF-κB/p50 transcription factors suggests its promising role in prostate cancer prevention. Inhibition of NF-κB activation could indirectly contribute to the pro-apoptotic action of α-tomatine on PC-3 cells as NF-κB controls the transcription of anti-apoptotic and cell proliferation genes, essential for the survival of cancer cells [44].

In summary, these results show that α-tomatine has good potentials as dietary phytochemical with pro-apoptosis activity. α-tomatine can be further explored for its therapeutic potential in the treatment of cancers where constitutive activation of NF-κB and apoptosis resistance remain a major concern in cancer chemotherapy. To establish the relevance of *in vitro* findings, further study is underway to investigate *in vivo* anti-tumor and NF-κB activities of α-tomatine using prostate xenograft cancer model.
